# Using Deep Learning Architectures for Detection and Classification of Diabetic Retinopathy

**DOI:** 10.3390/s23125726

**Published:** 2023-06-19

**Authors:** Cheena Mohanty, Sakuntala Mahapatra, Biswaranjan Acharya, Fotis Kokkoras, Vassilis C. Gerogiannis, Ioannis Karamitsos, Andreas Kanavos

**Affiliations:** 1 Department of Electronics and Telecommunication, Biju Patnaik University of Technology, Rourkela 769012, Odisha, India; cheena.mohanty@tat.ac.in; 2 Department of Electronics and Telecommunication Engineering, Trident Academy of Technology, Bhubaneswar 751016, Odisha, India; 3 Department of Computer Engineering-AI, Marwadi University, Rajkot 360003, Gujarat, India; 4 Department of Digital Systems, University of Thessaly, 41500 Larissa, Greece; fkokkoras@uth.gr (F.K.); vgerogian@uth.gr (V.C.G.); 5 Department of Graduate and Research, Rochester Institute of Technology, Dubai 341055, United Arab Emirates; ixkcad1@rit.edu; 6 Department of Informatics, Ionian University, 49100 Corfu, Greece; akanavos@ionio.gr

**Keywords:** convolutional neural networks, diabetic retinopathy, data balance, VGG16, XGBoost classifier, DenseNet 121

## Abstract

Diabetic retinopathy (DR) is a common complication of long-term diabetes, affecting the human eye and potentially leading to permanent blindness. The early detection of DR is crucial for effective treatment, as symptoms often manifest in later stages. The manual grading of retinal images is time-consuming, prone to errors, and lacks patient-friendliness. In this study, we propose two deep learning (DL) architectures, a hybrid network combining VGG16 and XGBoost Classifier, and the DenseNet 121 network, for DR detection and classification. To evaluate the two DL models, we preprocessed a collection of retinal images obtained from the APTOS 2019 Blindness Detection Kaggle Dataset. This dataset exhibits an imbalanced image class distribution, which we addressed through appropriate balancing techniques. The performance of the considered models was assessed in terms of accuracy. The results showed that the hybrid network achieved an accuracy of 79.50%, while the DenseNet 121 model achieved an accuracy of 97.30%. Furthermore, a comparative analysis with existing methods utilizing the same dataset revealed the superior performance of the DenseNet 121 network. The findings of this study demonstrate the potential of DL architectures for the early detection and classification of DR. The superior performance of the DenseNet 121 model highlights its effectiveness in this domain. The implementation of such automated methods can significantly improve the efficiency and accuracy of DR diagnosis, benefiting both healthcare providers and patients.

## 1. Introduction

The early detection and diagnosis of diabetes-related diseases are crucial in any healthcare system, as they enable improved treatment and care. Diabetes often coexists with various comorbidities in many patients. It is a metabolic disease characterized by elevated blood sugar levels; and, over time, it affects multiple organs in the human body [1]. In 2019, an estimated 463 million people worldwide were affected by diabetes [2]. Developing countries, such as China and India, are currently experiencing a diabetes epidemic [3]. According to the International Diabetes Federation [4], China, India, and the USA were the top three nations with the highest rates of diabetes in 2019; this trend is projected to continue from 2030 to 2045, with China and India bearing the greatest burden of the disease. Additionally, diabetes appears to be more prevalent in men (9.0%) than in women (7.9%) [5].

As previously mentioned, diabetes has a significant impact on various organs of the body, including the eyes. The ocular manifestation of diabetes is known as diabetic retinopathy (DR). DR primarily affects the eye retina, which is responsible for capturing light and transmitting signals to the brain. Like other parts of the body, the retina is also supplied with blood vessels. The elevated blood glucose levels in diabetes can lead to damage and alteration of the retinal blood vessels, resulting in fluid leakage [6]. Consequently, these pathological changes can lead to vision loss and, in some cases, permanent visual impairment [7]. Given these implications, DR is a highly significant and serious disease.

**Figure 1:**
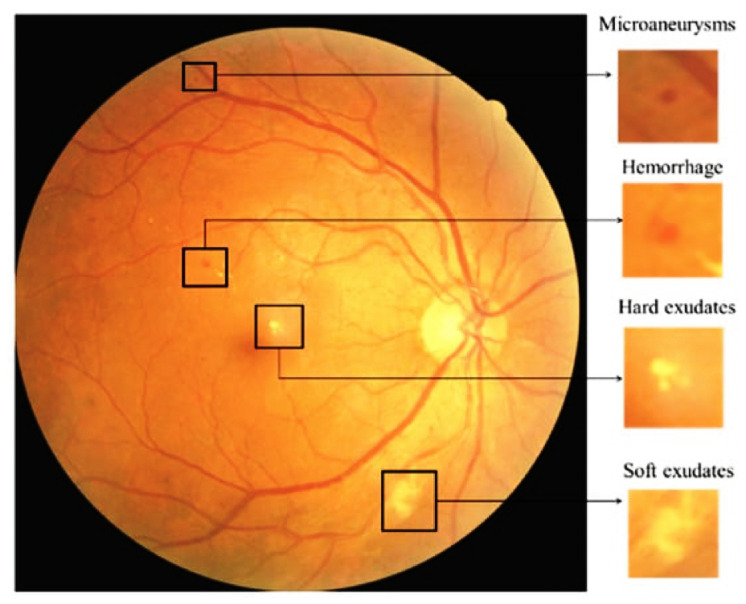
Diabetic retinopathy.

According to statistics [4], out of the 463 million individuals diagnosed with diabetes worldwide, approximately 30% also experience DR. This disease demonstrates a progressive nature, highlighting the importance of timely diagnosis and treatment to prevent permanent vision loss [8]. DR can be categorized into two subtypes: nonproliferative DR (NPDR) and proliferative DR (PDR). NPDR encompasses lesions such as microaneurysms and exudates [9]. Patients with DR exhibit various signs, as illustrated in Figure 1.

Regular screening of the retina is crucial for detecting DR, especially considering its asymptomatic nature in the early stages. Ophthalmologists typically rely on manual grading for this purpose. During the screening process, the patient’s pupil is dilated using specific eye drops, enabling the ophthalmologist to examine the retina using specialized lenses and lighting techniques. However, it is important to note that this procedure demands special expertise and experience to ensure accurate diagnosis.

With advancements in healthcare technologies, computer-aided diagnosis (CAD) systems have emerged as valuable tools for the rapid diagnosis of various diseases, including DR [10]. These systems offer cost-effective methods for retina screening; specifically, they can be extensively employed in the analysis of color fundus images for DR diagnosis. CAD systems enable experts to differentiate patients requiring more intensive treatment from those with less severe forms of the disease [11]. The underlying concept of CAD systems involves the expedited diagnosis of DR by analyzing features such as vessel segmentation, optic disk characteristics, and lesion segmentation from color fundus images. Subsequently, these images can be classified using various classifiers [12].

The evolution of artificial neural networks (ANNs) has paved the way for the development of more advanced machine learning (ML) techniques, such as deep learning (DL). In today’s healthcare systems, the role of artificial intelligence (AI) has become increasingly vital [13]. DL approaches, specifically in the field of medical image analysis and processing, have demonstrated their effectiveness [14,15]. DL has been successfully applied in not only the detection of DR but also ivarious other diseases, including the detection of COVID-19 [16]. DL approaches excel in extracting intricate patterns from data by leveraging filters and utilizing the given dataset to its full potential. The robustness of these models stems from their ability to handle vast amounts of data by employing deep neural networks [17].

DL models and, in particular, convolutional neural networks (CNNs), have emerged as effective solutions for DR classification problems, as supported by several studies [18,19,20]. CNNs make use of various pretrained models, including VGG16 [21], AlexNet [21], ResNet50 [22], and Inception-v3 [22]. The primary advantage of these models lies in their utilization of transfer learning [23,24,25], which allows models that have been previously trained on specific tasks to be repurposed for classification or detection in the context of another problem. This approach is particularly applicable in DL models, which often handle extensive datasets for training purposes.

The classification problems related to DR can be broadly categorized into two types: binary and multiclass. Binary classification focuses on distinguishing between a diseased retina and a healthy retina in color fundus images, as supported by the research [26,27]. On the other hand, multiclass classification approaches aim to grade the images into five different categories: Class 0—non DR, Class 1—mild DR, Class 2—moderate DR, Class 3—severe DR, and Class 4—proliferative DR [28,29]. Various publicly available datasets of retina images have been utilized in DL approaches for DR detection and diagnosis. Some notable examples include the DRIVE dataset [30], STARE [31], EyePACS dataset [32], ROC dataset [33], and Messidor dataset [34].

In this paper, we present our study focusing on experimentation with two powerful deep neural network models for the purpose of DR detection and classification. We conducted our experiments using the APTOS 2019 Blindness Detection dataset [35]. In both models, we employed the technique of transfer learning by utilizing pretrained feature detectors and training only the classifier component. More specifically, the two deep neural network approaches we utilized in our study were as follows:

- Hybrid model: This model is a combination of the VGG16 architecture, as the feature detector; and the XGBoost algorithm, as the classifier. By leveraging the strengths of both the DL architecture and the gradient boosting classifier, we aimed to enhance the overall performance of the system.
- DenseNet 121 Model: This model is based on the DenseNet 121 architecture, which is known for its dense connectivity pattern and efficient feature extraction capabilities. We employed this model to further explore its effectiveness in the context of DR detection and classification.

Furthermore, in order to enhance the performance these two models, we took steps to address the imbalanced nature of the selected dataset and applied preprocessing techniques to the considered images. In particular, to ensure a balanced representation of different classes, we trained the classifiers using a dataset that had been appropriately balanced. Additionally, we employed various preprocessing methods to enhance the quality and suitability of the images for our models’ training and evaluation processes.

The rest of this paper is organized as follows: Section 2 discusses recent research related to the detection, gradation, and classification of DR, providing an overview of relevant studies and approaches. Section 3 describes the dataset used in this study, specifically the APTOS 2019 Blindness Detection dataset, including its characteristics and relevant details. Section 4 presents the proposed methods, detailing the preprocessing steps applied to the images, introducing the hybrid model (combining VGG16 as the feature detector and XGBoost as the classifier) and the DenseNet 121 model. It also provides the experimental details, including software and parameter settings. Section 5 discusses the obtained results, analyzing the performance and accuracy of the proposed models and comparing them with existing methods. Finally, Section 6 concludes the paper, summarizing the key findings, suggesting future directions, and highlighting the contributions of this research.

## 2. Related Studies

Automated systems have emerged as potential solutions to facilitate early diagnosis and prevent permanent blindness in DR cases, eliminating the challenges associated with manual grading, which requires specialized expertise and can be burdensome for patients. To address these problems, researchers have explored various approaches for the classification of DR. This section presents a brief survey of models employed in this research field.

Rocha et al. [36] focused on addressing challenges in medical image analysis, including low contrast, poor lighting, and noise levels. Their study utilized the VGG16 network to classify retina fundus images into relevant categories. They employed publicly available datasets such as DDR, IDRiD, and EyePACS/Kaggle for image classification. The preprocessing steps included resizing the images and removing those with low contrast, followed by data augmentation, class balancing, hyperparameter adjustment, and image classification using the VGG16 network. Among the three databases, DDR exhibited the best performance, as measured via accuracy, precision, specificity, sensitivity, and the F1 score.

In their study, Khan et al. [37] focused on the challenge of reducing the model training time and convergence time in DR classification. They introduced a spatial pyramid pooling layer and network-in-network concepts alongside the VGG16 model. Preprocessing steps, such as resizing, cropping, normalization, and augmentation, were applied to the fundus retina images. The VGG16 network, spatial pyramid pooling layer, and network-in-network were stacked together. The spatial pyramid pooling layer connected the last convolutional layer of VGG16 with its first fully connected part, addressing concerns regarding cropping and information loss. The network-in-network layer was added on top to capture the nonlinear patterns within the datasets. The network-in-network part was initialized using the Xavier method, and fine-tuning was performed on the fully connected layers of VGG16. The results showed an AUC of 0.95 with 52% fewer parameters on the Kaggle dataset, and comparisons were made with the methods of other researchers.

Al-Antary et al. [38] proposed a CNN called multiscale attention network (MSA-Net) for detecting retina damage while dealing with high-level features. The authors utilized a multilevel and multiscale representation approach. Initially, the retina images were preprocessed using the APTOS and EyePACS datasets. A RESNET was employed as an encoder for feature extraction and to address the vanishing gradient issue. The extracted features encompassed local features and semantic information. To integrate these features, two multilevel representations were employed, combining mid-level and high-level features. A multiscale representation was utilized to ensure a uniform size, as the resolution of the features varied. The MSA-Net was then introduced to prioritize the relevant parts and differentiate the abnormalities in the retina images. The method achieved impressive performance on the APTOS database, with 98.1% accuracy, 98.3% sensitivity, 98.2% specificity, and an F1 score of 0.982, outperforming the other models. On the EyePACS database, the approach achieved an accuracy of 87.5%, sensitivity of 90.6%, specificity of 78.7%, and an F1 score of 0.767.

In [39], the authors proposed a hybrid model called E-DenseNet for the early diagnosis of DR. The motivation behind this model was to address research challenges in using a CNN for DR detection from retina images. Conventional CNNs may not accurately distinguish different types of lesions with distinct features. Hence, the E-DenseNet model was developed by stacking the Eyenet model on top of the DenseNet model, creating a customized hybrid architecture. The model was evaluated on four different datasets (EyePACS, IRiRD, Messidor, and APTOS 2019) spanning from 2006 to 2019 for detecting and classifying different grades of DR. The E-DenseNet model achieved impressive performance with an average accuracy of 91.2%, specificity of 69%, sensitivity of 96%, dice similarity coefficient of 92.45%, quadratic kappa score of 0.883, and a calculation time of 3.5 min.

Furthermore, Das et al. [40] conducted a comprehensive review of over one hundred research papers focused on the diagnosis of DR. The review highlighted various ML methods and their associated challenges in DR detection and diagnosis. It emphasized the superiority of DL architectures over traditional ML approaches in terms of feature extraction and image classification. In another study by Shaila et al. [41], a DL CNN model incorporating ResNet and VGG16 was developed for early DR detection. Texture analysis was performed on both balanced and imbalanced Kaggle datasets, and a combination of DL models was used for classification. The results demonstrated the model’s ability to accurately classify different stages of DR compared with other methods. Another approach involved the development of an intelligent system using case-based reasoning, as presented in Barman et al. [42]. This system employs retina image processing, feature extraction, and similarity-based case retrieval using the Euclidean distance measure to detect DR. All these studies highlight the effectiveness of DL models and the application of advanced techniques such as texture analysis and case-based reasoning in improving the accuracy and efficiency of DR detection and classification.

Challa et al. [43] proposed a deep All-CNN network for the diagnosis and grading of DR. In their study, they preprocessed images from the Kaggle dataset by applying Gaussian filters to enhance blending and remove retinal boundaries. The preprocessed images were then fed into the All-CNN network, which consists of ten convolution layers and a softmax layer for classification. The model achieved an accuracy of 86.64%, a loss of 0.46, and an average F1 score of 0.6318 across all five different stages of DR. Furthermore, other researchers [44,45] reviewed the contributions of numerous studies in the field of DR detection and classification, highlighting the implementation of both ML and DL models in these endeavors.

In recent years, several research studies have explored innovative techniques and applications in various fields. Federated learning has emerged as a promising approach to address the challenge of insufficient training data while maintaining data privacy. Authors [46] proposed a federated learning framework that allows multiple users to collaboratively train models locally without sharing sensitive data, enhancing performance and avoiding data privacy concerns. Additionally, in the domain of face swap deep fakes, Zhao et al. [47] introduced the conditional weighting transfer Wasserstein autoencoder, which enables effective knowledge transfer between multiple source domains. Biometrics, particularly face recognition, has attracted significant attention due to its uniqueness, stability, versatility, and difficulty to counterfeit, leading to its wide application [48]. Hyperspectral imagery has been recognized as valuable in remote sensing applications, including object classification, hyperspectral unmixing, anomaly detection, and change detection [49]. In the field of medical imaging, Ban et al. [50] proposed a novel 2D/3D registration model based on spatial histograms and tested it on X-ray and CT images. Other works [51,52] focused on Twitter sentiment analysis for the classification of user sentiments in tweets about COVID-19 on Twitter and implemented sentiment analysis using seven different deep learning models based on LSTM neural networks. Deep learning techniques have the potential to improve accuracy, and the work in [53] emphasizes the importance of employing the most up-to-date methods in the aviation industry. These references highlight the advancements and diverse applications of various techniques in different domains, contributing to the development of robust and effective solutions.

Table 1 provides an overview of the techniques proposed in relevant studies and summarizes the techniques employed in the current study.

## 3. Dataset Description

This section focuses on the dataset used in our study and describes how the dataset was balanced. The APTOS 2019 Blindness Detection Database [35] was employed, which consists of 3662 retinal images captured under various lighting conditions. The dataset was collected from the Aravind Eye Hospital in India. The retinal images in the dataset are categorized into five classes representing different severity levels of DR: Class 0 corresponds to non-DR, Class 1 corresponds to mild DR, Class 2 corresponds to moderate DR, Class 3 corresponds to severe DR, and Class 4 corresponds to proliferative DR, as outlined in Table 2. The distribution of the samples across these severity levels is presented in Table 3, indicating the number of images in each class. The dataset was balanced to ensure a sufficient representation of each severity level, which is crucial for effectively training and evaluating the models.

**Figure 2:**
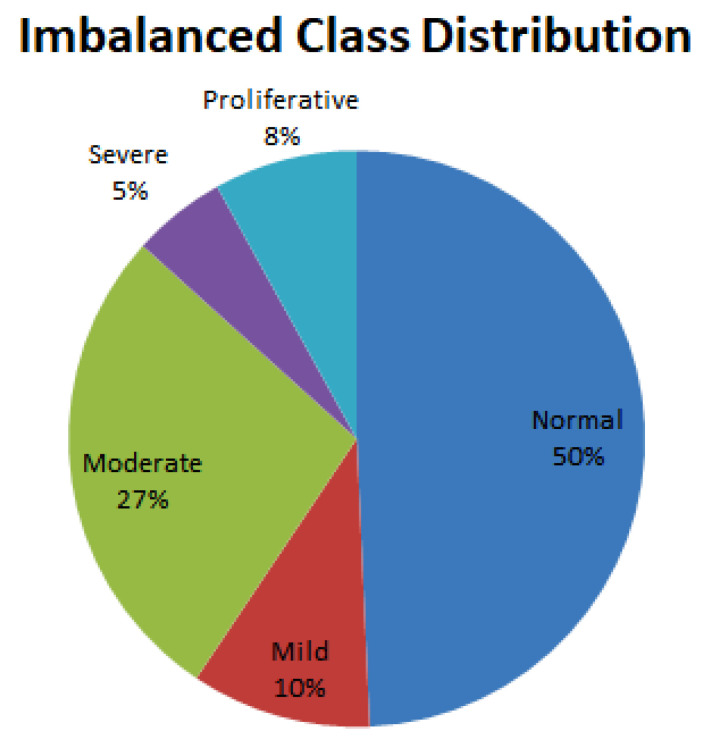
Imbalanced class distribution of the severity level of diabetic retinopathy.

The APTOS 2019 Blindness Detection database exhibits a significant class imbalance, as illustrated in Figure 2. To address this issue, dataset-balancing techniques [54,55] were applied as a crucial preprocessing step. Balancing the training and testing datasets involved adjusting the ratios to minimize the disparities between the classes. This ensured that each class had a more equal representation, enabling more effective training and evaluation of the models.

**Figure 3:**
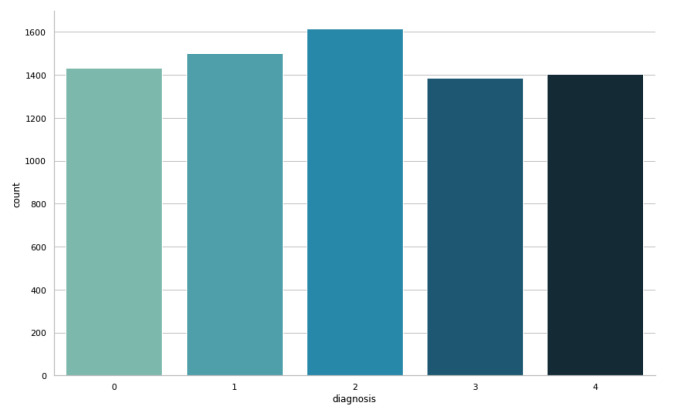
The balanced training dataset.

**Figure 4:**
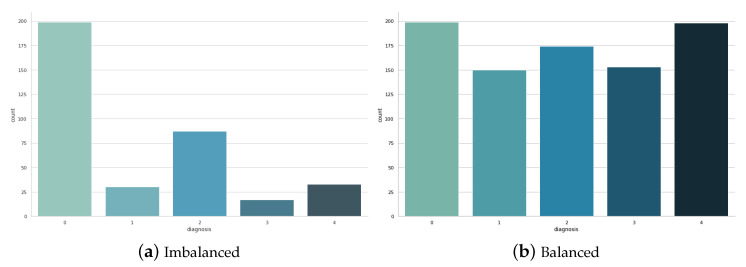
Imbalanced and balanced testing datasets.

Figure 3 displays the balanced training dataset, where each class is represented by an approximately equal number of samples. On the other hand, Figure 4a,b illustrate the imbalanced and balanced testing datasets, respectively. In the imbalanced testing dataset (Figure 4a), the class distribution reflects the original dataset; while in the balanced testing dataset (Figure 4b), the classes were adjusted to achieve a more balanced representation.

## 4. Methodology

In this section, we provide a detailed description of the proposed method. Our approach involves the implementation and experimentation of two different deep learning (DL) models: a hybrid model based on the combination of VGG16 and XGBoost classifier and a DenseNet 121 model.

The hybrid model was designed to leverage the strengths of both VGG16, a popular convolutional neural network (CNN) architecture known for its effectiveness in feature extraction; and XGBoost, a powerful gradient boosting algorithm widely used for classification tasks. By combining these two models, we aimed to enhance the overall performance of the system.

The second model, DenseNet 121, is a deep CNN architecture known for its dense connectivity pattern, which allows for efficient information flow between layers. This model has shown promising results in various image classification tasks and was well suited for our objective of DR detection and classification.

### 4.1. Image Preprocessing

**Figure 5:**
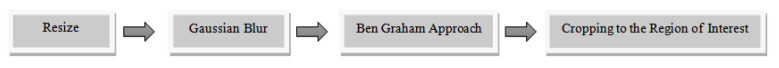
Various image preprocessing techniques.

The images in the considered dataset were collected from rural parts of India under diverse conditions, leading to a lack of uniformity among them. Utilizing these images in their raw form would not have yielded the desired results. Therefore, preprocessing was necessary to enhance the images before feeding them into the neural network model. The application of various preprocessing techniques, as depicted in Figure 5, aimed to standardize and optimize the images, ensuring improved quality and facilitating accurate analysis and classification.

**Figure 6:**
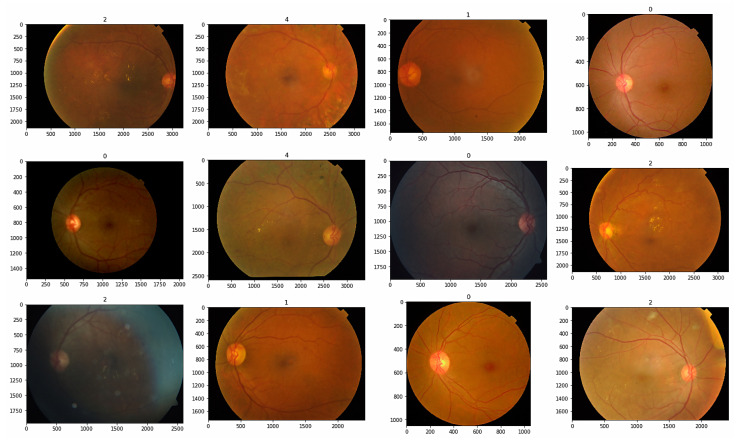
Input sample images.

Figure 6 showcases a selection of sample images included in the dataset.

**Figure 7:**
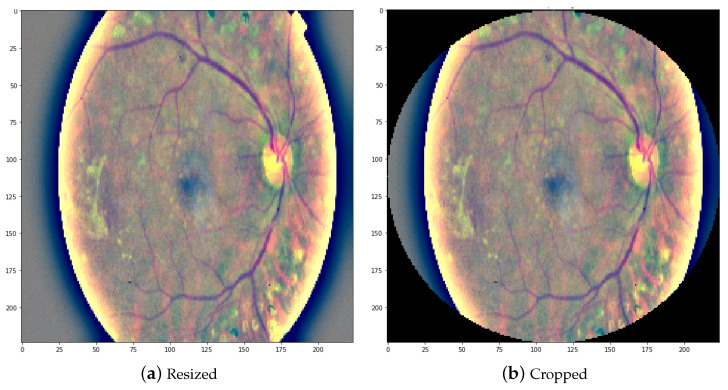
A resized input image and an image cropped to the region of interest.

The size of the images in the dataset was not uniform, as they were collected from different places. To standardize the input, a series of preprocessing steps was applied to the images:

- First, all images were uniformly resized to a fixed dimension of 224 × 224 pixels, as shown in Figure 7a. This resizing step ensured that all images had the same size, facilitating consistent analysis.
- Additionally, a Gaussian blur filter was applied to reduce noise and enhance image quality.
- Finally, the Ben Graham procedure [56] was utilized to further improve image quality and accuracy. This involved cropping the images to their region of interest, as depicted in Figure 7b.

These preprocessing steps enable the neural network models to receive standardized and optimized input images, and thus improving the overall performance of the system.

### 4.2. Modeling

To detect unhealthy retina images, we developed two distinct DL models that are capable of accomplishing this task. The first model is a hybrid model, while the second model is based on the DenseNet 121 architecture. In this section, we discuss each model in detail, starting with the hybrid model and then proceeding to the DenseNet 121 model.

#### HybridModel: VGG16 and XGBoost Classifier

**Figure 8:**
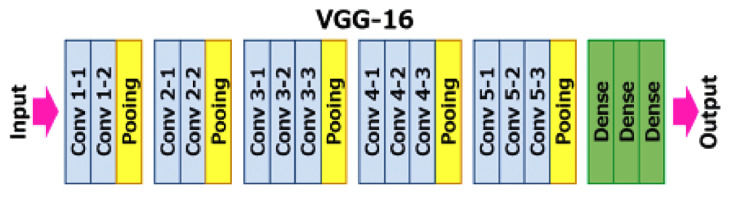
VGG 16 architecture.

The VGG16 and XGBoost classifiers were combined to create a hybrid model for detection and classification of diabetic retinopathy (DR). The VGG16 network, which is a powerful pretrained convolutional neural network (CNN), was used as the base model for image classification [57,58]. It has 16 layers of processing, including convolutional and max pooling layers [59], as depicted in Figure 8.

To enhance the performance of the VGG16 model, an XGBoost classifier was employed. XGBoost is a boosting decision tree classifier that optimizes a cost function through gradient descent [60]. Unlike traditional ensemble classifiers that adjust the weights of the training set [61], XGBoost boosts a weak model to improve its predictive power.

In the hybrid model, the output from the VGG16 network is fed as the input to the XGBoost classifier, allowing for a combination of the powerful feature extraction capabilities of VGG16 with the gradient boosting capabilities of XGBoost. This integration aims to enhance the classification accuracy and performance of the model in detecting and classifying DR.

More specifically, the VGG16 CNN consists of 13 convolutional layers and 3 fully connected layers. Each convolutional layer has a kernel size of 3 × 3 and uses ReLU activation. The number of channels increases gradually from 64 to 512 in the deeper layers. Max pooling layers with a 2 × 2 window and stride of 2 are applied after certain convolutional blocks to downsample the feature maps.

After the feature extraction process with VGG16, the extracted features are fed into the XGBoost classifier. XGBoost is a gradient boosting algorithm that uses an ensemble of decision trees. The decision trees are sequentially trained, with each subsequent tree trying to correct the mistakes made by the previous trees. The number of decision trees and other hyperparameters of the XGBoost classifier were optimized through cross-validation.

To combine the VGG16 CNN and XGBoost classifier, we used a two-step approach. First, we trained the VGG16 CNN on the training data and obtained the output features from the last fully connected layer. These features served as the input to the XGBoost classifier, which was separately trained using the labeled data. During inference, the input image was first passed through the VGG16 CNN to extract features, and then these features were used as input to the trained XGBoost classifier for the final prediction. By combining the strengths of both deep learning and gradient boosting techniques, the hybrid model aims to leverage the feature extraction capabilities of CNNs and the powerful ensemble learning of XGBoost to improve the overall classification performance.

### 4.3. DenseNet 121 Model

**Figure 9:**
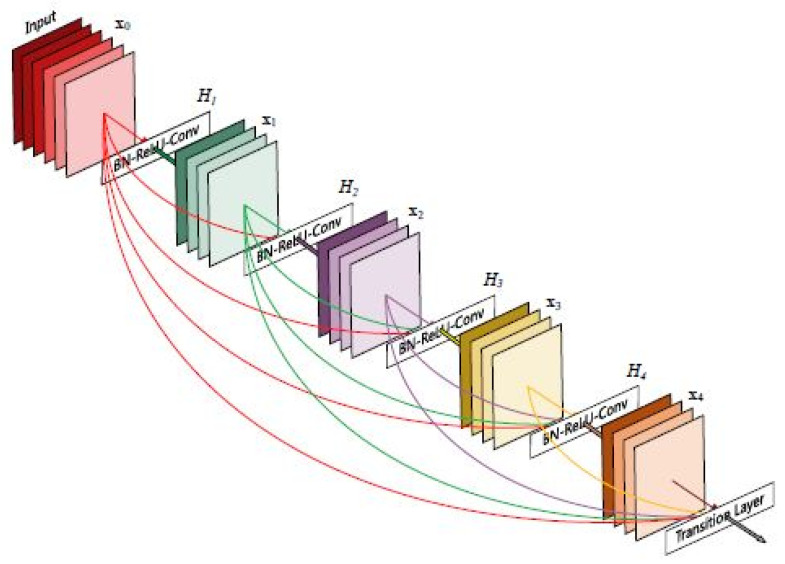
DenseNet model architecture.

The DenseNet is a type of convolutional neural network (CNN) that enables deeper network architectures by connecting each layer to every other layer in a feed-forward fashion [62]. In the DenseNet model, each layer receives inputs from all preceding layers and passes its feature maps to all subsequent layers, resulting in a dense connectivity pattern. This dense connectivity allows for efficient information flow and promotes feature reuse throughout the network [63]. The architecture of the DenseNet 121 model is illustrated in Figure 9.

The DenseNet architecture consists of basic convolutional and pooling layers, dense blocks, and transition layers. The model begins with a convolutional block that applies a sliding window of size 7 × 7 to the input image, producing 64 output layers/filters. This block uses a stride of 2, resulting in a downsampled feature map. It is followed by a max pooling layer with a 3 × 3 sliding window and a stride of 2, further reducing the spatial dimensions of the feature map.

Within the DenseNet architecture, there are multiple dense blocks, each consisting of a sequence of operations. The convolutional blocks within each dense block follow a specific pattern. They begin with a batch normalization layer to standardize the input, followed by a ReLU activation function to introduce nonlinearity, and then a Conv2D layer to perform the convolution operation. In DenseNet 121, this sequence of batch normalization, ReLU activation, and Conv2D is repeated 6 times in the first dense block, 12 times in the second dense block, 24 times in the third dense block, and 16 times in the final dense block.

The transition layers in DenseNet reduce the number of channels in the feature maps. The transition layers are placed immediately after each dense block. They consist of a 1 × 1 convolutional layer followed by a 2 × 2 average pooling layer with a stride of 2. This combination progressively reduces the number of channels by half from one dense block to the next. In DenseNet 121, the number of channels is reduced from 256 to 128, then to 64, and finally to 32.

Finally, in the DenseNet architecture, there is a global average pooling layer that performs spatial pooling across the entire feature map, resulting in a fixed-length vector representation [64]. This pooling operation aggregates the feature maps into a compact representation that captures the most salient information. Finally, a fully connected layer is used for classification, followed by a softmax activation function to generate the class probabilities.

### 4.4. Experimental Details

The considered models were trained and tested for binary classification using the TensorFlow [65] and Scikit-Learn [66] libraries in Python programming language. The Adam optimizer [67] was used for optimizing the training process. Table 4 shows the different parameter settings for the hybrid model and the DenseNet 121 model.

The initialization of hyperparameters is a critical aspect in deep learning models as it can significantly impact their performance and convergence. In our study, we followed the established practices for initializing hyperparameters based on the characteristics of the specific models employed.

The learning rate is a configurable hyperparameter used in the training of neural networks. It determines the amount that the weights are updated during each iteration, also referred to as the step size. The learning rate acts as a scale factor for the gradients computed during backpropagation, influencing the speed and quality of convergence. A higher learning rate can result in faster convergence, but it may also risk overshooting the optimal solution. Conversely, a lower learning rate can ensure more precise weight updates, but it may require more training epochs to reach convergence.

The batch size is another important hyperparameter that determines the number of samples processed before the model is updated. In each training iteration, the batch size specifies the subset of data samples used to compute the gradients and update the model’s parameters. Choosing an appropriate batch size involves balancing computational efficiency and the quality of weight updates. Smaller batch sizes provide more frequent updates, leading to faster convergence but with higher computational overhead. On the other hand, larger batch sizes can leverage parallelism and optimize computational efficiency but may result in less frequent weight updates and potentially slower convergence.

Furthermore, the number of epochs defines the number of complete passes through the training dataset during training. Each epoch consists of multiple iterations, where the model updates its weights based on the gradients computed from the batched data. The number of epochs is typically set based on the convergence behavior of the model and the desired level of training. More complex tasks or models may require a greater number of epochs to reach convergence, while simpler tasks or models may converge faster.

The initialization basis for these hyperparameters was carefully considered in our study, taking into account the characteristics of the deep learning models and the requirements of the diabetic retinopathy classification task.

The models were evaluated using the accuracy metric, which is calculated as follows:

(1) $$Accuracy=\frac{TP+TN}{TP+TN+FP+FN}$$

where TP represents the true positives (the number of correct predictions of unhealthy retina images), TN represents the true negatives (the number of correctly predicted healthy retina images), FP represents the false positives (the number of healthy retina images incorrectly predicted as unhealthy), and FN represents the false negatives (the number of unhealthy retina images incorrectly predicted as healthy). This metric provides an overall measure of the models’ performance in correctly classifying the retina images.

## 5. Results and Discussion

**Figure 10:**
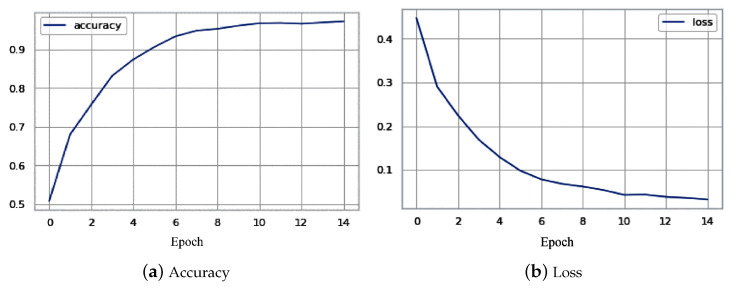
Accuracy and loss function of DenseNet 121 CNN model.

The models were trained and tested on the APTOS dataset, with 80% of the data used for training and 20% used for testing. The training process was conducted over 50 epochs. The hybrid model achieved an output accuracy of approximately 80%, while the DenseNet 121 model achieved an impressive overall accuracy of 97.30%. The accuracy and loss curves for the DenseNet 121 model are shown in Figure 10a,b, respectively.

**Figure 11:**
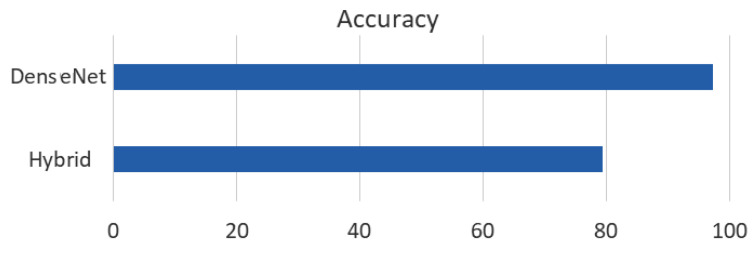
Comparison between the hybrid and the DenseNet 121 model.

The performance of the two proposed models, the hybrid model and the DenseNet 121 model, was evaluated and compared in terms of accuracy. The results of the comparison are presented in Figure 11. From the figure, it can be observed that the DenseNet 121 model outperformed the hybrid model in terms of accuracy. The DenseNet 121 model achieved an accuracy of 97.30%, while the hybrid model achieved an accuracy of approximately 80%.

The higher accuracy of the DenseNet 121 model can be attributed to its dense connectivity pattern, which allows for effective information propagation throughout the network. This enables the model to capture and utilize important features from all preceding layers, leading to more accurate predictions.

The performance of the proposed hybrid model and DenseNet 121 model was compared with that of several existing methods using different datasets, including APTOS, EyePACS, and Messidor. The comparison of the models with other researchers’ methods is presented in Table 5. The proposed hybrid model performed better and obtained an accuracy of 79.50% compared with an accuracy of 75.61% of CNN [68] on the APTOS 2019 Blindness Detection Kaggle Dataset. Moreover, the DenseNet 121 model achieved a higher accuracy score of 97.30% than other existing techniques, such as Inception V3 [69], CNN [70], Inception ResNet V2 [71], and GoogleNet [72], on the APTOS 2019 Blindness Detection dataset.

### Discussion

While our approach in this study incorporates the VGG model as a foundation, it is important to note that we made specific structural updates and modifications to adapt it to the task of medical image classification, particularly for diabetic retinopathy detection.

Medical image classification poses unique challenges and requires specialized considerations due to the complexity and intricacy of medical imaging data. The specific structural updates we made to the VGG model for medical image classification include:

- Preprocessing: Medical images often require specific preprocessing steps such as normalization, resizing, and data augmentation techniques tailored to the characteristics of the medical imaging data. These preprocessing steps help with improving the robustness and generalization of the model.
- Transfer learning: Given the limited availability of labeled medical image datasets, transfer learning becomes crucial. We leveraged transfer learning by initializing the VGG model with pretrained weights on large-scale image datasets and fine tuning it on our specific medical image dataset. This transfer of knowledge from general image classification tasks to the medical domain helps with learning relevant features and patterns.
- DenseNet architecture: In addition to the VGG model, we also employed the DenseNet architecture, which has shown promising performance in various medical image analysis tasks. DenseNet introduces dense connections between layers, facilitating feature reuse and gradient flow throughout the network. This architecture helps with capturing more intricate details and dependencies within the medical images.
- Class imbalance handling: Class imbalance is a common challenge in medical image classification tasks, where certain classes have significantly fewer samples than others. To address this, we employed techniques such as data augmentation, class weighting, and sampling strategies to balance the class distribution during training, ensuring that the model effectively learned from all classes.

These specific structural updates and adaptations are essential for enhancing the performance and relevance of the neural network models in the context of medical image classification. By tailoring the architecture and incorporating domain-specific considerations, we can effectively leverage the power of advanced neural network models to accurately and reliably analyze medical images.

## 6. Conclusions and Future Work

The early detection of diabetic retinopathy is crucial in preventing vision loss caused by diabetes mellitus. Computer-aided diagnosis (CAD) systems have significantly simplified the process of regular eye screening for diabetic patients [73]. With the advancements in CAD systems, deep learning neural networks, such as the ones examined in this study, have emerged as powerful tools for retinal image classification. In this study, we investigated two deep learning models, namely, a hybrid model (a combination of VGG16 and XGBoost Classifier) and a DenseNet 121 model. An essential step in our approach was to balance the training and testing datasets of the APTOS 2019 Blindness Detection database. While the hybrid model did not yield satisfactory results, the DenseNet 121 model demonstrated superior classification accuracy. We also compared our proposed models with existing methods on the same dataset, and the results revealed that the DenseNet 121 model achieved an impressive accuracy of 97.30%, outperforming all other compared architectures. Although the hybrid model achieved an accuracy of 79.50%, it still performed better than the CNN model [68]. Furthermore, the DenseNet 121 model exhibited fast classification capabilities, making it suitable for real-time medical applications.

In terms of future work, several techniques can be explored to further enhance the performance of the proposed models in retinal image classification tasks [74]. One significant contribution would involve the development of an application that can assist medical experts and even patients in the early detection of diabetic retinopathy. Such an application would not only prevent vision loss but also save valuable therapy time and costs [75]. By leveraging the power of deep learning models such as DenseNet 121, this application could provide efficient and reliable diagnoses, benefiting individuals at risk of developing diabetic retinopathy.

## Figures and Tables

**Table 1:** Overview of relevant approaches and the proposed techniques.

Paper	Proposed Techniques
[36]	Removal of low-contrast images, data augmentation, and classification using VGG16 network.
[37]	Utilization of spatial pyramid pooling layer and network-in-network layer in conjunction with VGG16 network.
[38]	Introduction of a multiscale attention network (CNN) capable of detecting damages while handling high-level features.
[39]	Development of E-DenseNet, an ensemble model combining EyeNet and DenseNet architectures.
[41]	Texture analysis performed on balanced and imbalanced datasets using various CNN models.
[42]	Implementation of an expert system utilizing case-based reasoning with retina image processing and feature extraction.
[43]	Adoption of an All-CNN network consisting of ten convolution layers and a softmax layer.
Current approach	Two DL models are examined: a hybrid model (a combination of VGG16 and XGBoost Classifier) and one based on the DenseNet 121 architecture.

**Table 2:** Severity levels of diabetic retinopathy.

**Class**	0	1	2	3	4
**Classification**	Non-DR	Mild DR	Moderate DR	Severe DR	Proliferative DR

**Table 3:** Distribution of samples as per severity level of diabetic retinopathy.

Severity Level	Number of Samples
Class 0 (normal)	1805
Class 1 (mild)	370
Class 2 (moderate)	999
Class 3 (severe)	193
Class 4 (proliferative)	295

**Table 4:** Parameters for the hybrid and DenseNet 121 models.

	Hybrid Model	DenseNet 121 Model
**Batch Size**	16	32
**Initial Learning Rate**	0.01	0.01
**Minimum Learning Rate**	0.0001	0.00005
**Epochs**	50	50

**Table 5:** Performance comparison of different architectures with the proposed models.

Paper	Dataset	Architecture	Accuracy
[69]	APTOS	Inception V3	82
[70]	APTOS	CNN	94.44
[71]	APTOS	Inception Res Net V2	82.18
[72]	APTOS	Google Net	97
[68]	APTOS	CNN	75.61
Present work	APTOS	Hybrid	79.50
Present work	APTOS	DenseNet 121	97.30
